# Cardiac Implantable Electronic Device Infections in Patients with Renal Insufficiency: A Systematic Review and Meta-Analysis

**DOI:** 10.3390/diseases12100247

**Published:** 2024-10-10

**Authors:** Deepak Chandramohan, Prabhat Singh, Hari Naga Garapati, Raghunandan Konda, Divya Chandramohan, Nihar Jena, Atul Bali, Prathap Kumar Simhadri

**Affiliations:** 1 Department of Internal Medicine/Nephrology, University of Alabama at Birmingham, Birmingham, AL 35233, USA; rkonda@uabmc.edu; 2 Department of Nephrology, Christus Spohn Hospital, Corpus Christi, TX 78404, USA; drprabhatsingh@hotmail.com; 3 Department of Nephrology, Baptist Medical Center East, Montgomery, AL 36106, USA; drvenug@gmail.com; 4 Department of Internal Medicine/Infectious Disease, University of Texas Health, San Antonio, TX 78207, USA; chandramohan@uthscsa.edu; 5 Department of Internal Medicine/Cardiovascular Medicine, Trinity Health Oakland/Wayne State University, Pontiac, MI 48341, USA; niharjmd@gmail.com; 6 Department of Internal Medicine/Nephrology, Geisinger Health, Danville, PA 18702, USA; dr.atulbali@gmail.com; 7 Department of Nephrology, Advent Health/FSU College of Medicine, Daytona Beach, FL 32117, USA; prathap.simhadri@gmail.com

**Keywords:** cardiac implantable electronic device, renal insufficiency, chronic kidney disease, end-stage renal disease, device-related infections

## Abstract

**(1) Background:** Renal insufficiency is a risk factor for cardiac implantable electronic device (CIED) infection. **(2) Methods**: A comprehensive search was conducted from multiple electronic databases to identify studies. Using the random effects model, we calculated the pooled rates of CIED infection and their 95% confidence intervals. We also calculated the pooled odds ratios to determine the risk of CIED infections due to chronic kidney disease (CKD) and end-stage renal disease (ESRD). We utilized the Cochran Q and I2 statistics to detect and quantify heterogeneity. **(3) Results**: A total of 17 studies comprising 359,784 patients with renal insufficiency were added to the meta-analysis. Out of these, 263,819 were CKD patients and 89,617 were ESRD patients. The pooled rate of CIED infection in patients with CKD was 4.3% (95% CI: 2–8.8; I2: 95.7), and in patients with ESRD, it was 4.8% (95% CI: 2.6–8.7; I2: 99.4). The pooled risk of CIED infection in the CKD population was OR 2.5 (95% CI: 1.9–3.3; *p* < 0.001; I2: 21.1), and in the ESRD population, it was OR 2.4 (95% CI: 1.01–5.7; *p* = 0.046; I2: 88.8). ESRD was associated with higher mortality, OR 2.5 (95% CI: 1.4–4.4.8; *p* = 0.001; I2: 95). **(4) Conclusions**: The presence of renal insufficiency increases the number of CIED infections. In particular, patients with ESRD have an increased risk of mortality.

## 1. Introduction

Cardiac implantable electronic devices (CIEDs) are used for a multitude of reasons, such as chronotropic support, improving hemodynamics, and the prevention of sudden cardiac death [1]. The number of patients with cardiac implantable electronic devices has increased in the United States, and between 1993 and 2008, over 4.2 million CIED implantations were performed, representing an increase of 96% [2]. Left ventricular dysfunction is frequent in renal failure, and the incidence of sudden cardiac death increases with declining renal function, requiring the use of a pacemaker (PPM) or an implantable cardioverter–defibrillator (ICD) [3,4]. According to the US Renal Data System database (USRDS), cardiac arrhythmia and sudden cardiac death occur in up to 40% of end-stage renal disease (ESRD) patients on dialysis [5]. Infections of these devices in the general population have grown exponentially in the past years, from 1.45% in 2000 to 3.41% in 2012 [6]. Complications such as bacteremia and endocarditis have increased, especially in the presence of central venous catheters [7]. According to various studies, the risk of CIED infection is 1.5 times higher in patients with chronic kidney disease (CKD) and 2 times higher in patients with ESRD [8,9]. Device infections in patients with renal insufficiency carry high mortality. There are also associated increased healthcare costs and prolonged hospital stays [10,11].

Patients with renal insufficiency have been generally underrepresented in ICD clinical trials, and there are little data about the worldwide rates of CIED infections with renal insufficiency [3,12]. CIED infections can be either localized pocket infections or systemic infections [13,14]. Given the increased susceptibility to infections in people with ESRD, understanding the incidence, clinical outcomes, and risk factors for CIED infections in this population is critical for improving patient care. We performed a meta-analysis to investigate the risk of infection due to renal insufficiency and the infection rates in this population. We also analyzed the mortality risk of ESRD patients with CIED infections.

## 2. Materials and Methods

### 2.1. Literature Search Strategy

Using a combination of search terms a comprehensive search was conducted on PubMed/Medline, Embase, Cochrane Library, and Google Scholar from inception to February 2024. We used cardiac device-related terms such as “Cardiac device infections” OR “Pacemakers” OR “Implantable cardioverter-defibrillators” with infection-related terms such as “Infections” OR “Infection” OR “Complications” OR “Complication” along with outcome-related terms such as “Incidence” OR “Prevalence” OR “Clinical outcomes” OR “Clinical outcome” OR “Morbidity” OR “Mortality” OR “Risk factors” OR “Risk factor” and renal dysfunction-related terms “End-Stage Renal Disease” OR “ESRD” OR “End stage renal disease” OR “Chronic kidney disease” OR “CKD”. We also searched for relevant publications from the bibliographic sections of articles of interest. Details of the literature search are shown in Table S1 in the Supplementary Materials section.

### 2.2. Selection Criteria and Data Extraction

Two authors (D.C. and P.S.) independently reviewed the abstracts of the retrieved studies to determine their eligibility. After screening, the full-text publications were examined. The studies that met the inclusion and exclusion criteria were added, and any differences in opinion were settled through consensus or after consulting a third author (P.K.S.). The MOOSE (Meta-analyses Of Observational Studies in Epidemiology) checklist was followed (Supplementary Materials Table S3) [15]. The final studies were selected using the Preferred Reporting Items for Systematic Reviews and Meta-Analyses (PRISMA) (Supplementary Materials Table S4) [16]. The MOOSE and PRISMA checklists are shown in the Supplementary Materials section. The study protocol was registered in PROSPERO, registration number CRD42023400049.

The inclusion criteria were (1) patients ≥ 18 years old with CIED infection and CKD or ESRD; (2) studies that report the rates of CIED infection or odds ratio. The exclusion criteria were as follows: (1) pediatric studies; (2) studies reporting CIED infections in non-CKD or ESRD populations; (3) studies lacking relevant outcome data; (4) studies reporting outcomes of lead extraction; (5) studies reporting on lead extraction due to infection; (6) studies not in English; and (7) conference abstracts, case reports, case series, and review articles.

All of the authors extracted the data into a standardized form. The extracted data consisted of author details, years of study, demographic information, comorbidities, rates of infection, and other pertinent information. The extracted information was verified by two authors (D.C. and P.K.S.). If the studies had overlapping cohorts and patient data, we only included the studies with complete information.

### 2.3. Outcomes of Interest

The primary outcome was the CIED infection rate in patients with CKD and ESRD. The secondary outcomes were the risk of CIED infection due to renal insufficiency and mortality in ESRD.

### 2.4. Statistical Analysis

The mean and standard deviation were used to express continuous variables. Percentages, along with counts, were used to describe categorical variables. We assumed that all of the studies were selected from a random sample from a large pool of studies, and therefore, we used the random effects model [17,18]. The inverse variance random effects DerSimonian–Laird method was used to calculate the pooled outcomes and their 95% confidence intervals (CI) [19]. We conducted a meta-analysis of proportions to determine the rates of infection. Pooled odds ratios (OR) and 95% CI were calculated to assess the risk of infection due to renal insufficiency and to determine mortality due to ESRD. A *p*-value of <0.05 was considered significant for the pooled OR. Forest plots were later created to visualize the statistical analysis results [20].

The determination of heterogeneity was performed through two methods. First, we used the Cochran Q statistic to detect if the null hypothesis holds true. The null hypothesis is that all the studies had the same effect size. If this were true, the value of Q would be equal to the degrees of freedom. The presence of a greater value for Q than the degree of freedom would reject the null hypothesis [21]. Due to a lack of statistical power, the alpha threshold for this test is commonly set at 0.10 rather than 0.05. Second, we use the I2 statistic to measure and quantify heterogeneity. The I2 statistic measures the proportion of variability in effect sizes that is not due to sampling error. If heterogeneity is detected using I2, it is quantified as low if values are <30%, moderate if values are between 31 and 60%, substantial if values are between 61 and 75%, and considerable if values are >75% [22]. Publication bias was ascertained by visual inspection of the funnel plot. In the presence of asymmetry, we used the Egger regression test to assess bias further. A one-tailed *p*-value of <0.05 would suggest the presence of publication bias. Statistical analysis was conducted using Comprehensive Meta-Analysis software, version 4 (Biostat, Englewood, NJ, USA) [23].

### 2.5. Quality Assessment and Risk of Bias

Quality assessment was performed using the Newcastle–Ottawa scale (NOS) for cohort studies. NOS evaluates studies based on selection, comparability, and outcome. The selection domain assesses the study sample selection process. The comparability domain assesses the controls in the study, while the outcome domain assesses the follow-up process, including its adequacy. These three domains are further divided into eight subdomains. Studies are scored between 0–9. Studies with a score >5 are high quality, those with a score of 3–5 are medium quality, and those with a score <3 are low quality [24,25]. Two authors (D.C. and P.K.S.) independently performed the quality assessment. When the scoring had discrepancies, it was resolved in a similar manner to the study selection process.

### 2.6. Ethical Approval and Consent to Participate

Ethics approval was not required for our meta-analysis because the data are accessible to the public.

## 3. Results

### 3.1. Search Results

**Figure 1:**
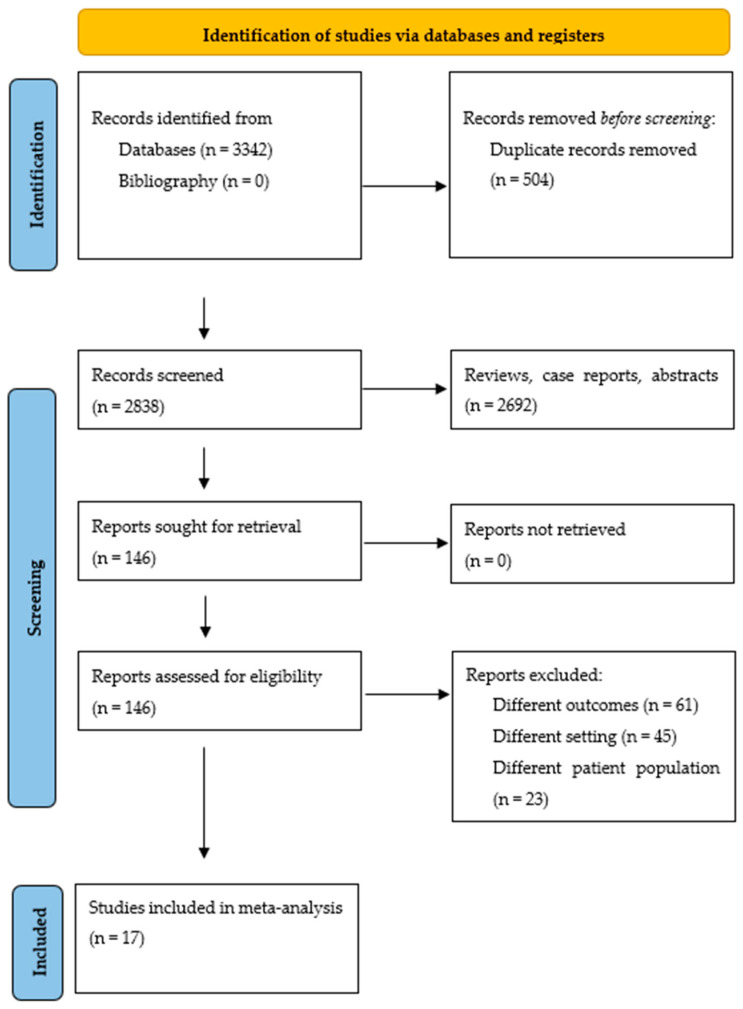
Study selection process according to the Preferred Reporting Items for Systematic Reviews and Meta-AnalysEs statement.

The literature search yielded 3342 citations. After eliminating duplicates, case reports, abstracts, and review articles, 2838 citations were screened through abstract review, and 146 articles were selected for full-text review. Using the pre-specified inclusion and exclusion criteria, 129 articles were later eliminated. Finally, 17 studies were included in the meta-analysis. Figure 1 displays the flowchart of the study selection process. Studies with cohort overlap are shown in the Supplementary Materials, Table S2.

### 3.2. Study Characteristics

Fifteen of the included studies were retrospective [1,8,26,27,28,29,30,31,32,33,34,35,36,37,38]. There was one prospective single-center study by Romeyer-Bouchard et al. [39] and one cluster randomized cross-over trial by Krahn et al. [40]. There were seven studies that reported CIED infections in the ESRD population [26,29,31,32,34,35,36], one study reported CIED infections in CKD (28), and nine studies reported both ESRD and CKD patients [1,8,27,30,33,37,38,39,40]. Studies by Charytan et al. and Jeong et al. reported cardiac device infections in ESRD-only cohorts [29,32]. A total of seven studies were population-based [1,26,29,33,34,35,40]. Modi et al. investigated 314,418 patients with renal insufficiency from the National Inpatient Sample (NIS) database; this was the largest cohort in the meta-analysis [1]. There were six studies conducted in the United States [1,8,26,29,35,37], three in Taiwan [30,33,38], and the rest from other countries. Three studies [8,27,38] reported using the Modification of Diet in Renal Disease (MDRD) equation to estimate glomerular filtration rate (GFR), while Tompkins et al. used the Cockcroft–Gault equation [37]. There were considerable differences in the reported rates of infection among patients with CKD and ESRD. Calderón-Parra et al. reported a high number of infections, 9 out of 33 CKD patients [28]. Romeyer-Bouchard et al. reported a high number of infections in 3 out of 8 ESRD patients [36]. The summary of the included studies is shown in Table 1.

### 3.3. Patient Characteristics

The total number of patients with renal insufficiency was 359,784, out of which 263,819 had CKD and 89,617 had ESRD. The mean age of patients with renal insufficiency was 68.6 years (95% CI: 66.4–70.8; I2: 99.7). Among these patients, hypertension was present in 69.7% (95% CI: 31.6–92), diabetes mellitus was present in 50.5% (95% CI: 44.6–56.4), congestive heart failure (CHF) was present in 65% (95% CI: 41.5–82.9), coronary artery disease (CAD) was present in 45.6% (95% CI: 22.8–70.3), atrial fibrillation was present in 35.4% (95% CI: 32.9–37.9), and 21.7% (95% CI: 7.2–49.7) were reported to be on anticoagulation. Among the studies investigating patients in the United States, Whites made up 52.7% (95% CI: 39.2–65.8) and Blacks made up 27.8% (95% CI: 26–29.7).

The pooled percentage of patients with PPM was 85.5% (95% CI: 25.7–99), that of ICD was 44.5% (95% CI14.9–78.5), and that of cardiac resynchronization therapy (CRT) was 13.6% (95% CI: 7.1–24.7). The mean follow-up period was 45.4 months (95% CI: 35.7–55; I2:96.6). The patient characteristics are shown in Table 2.

### 3.4. Outcomes

**Figure 2:**
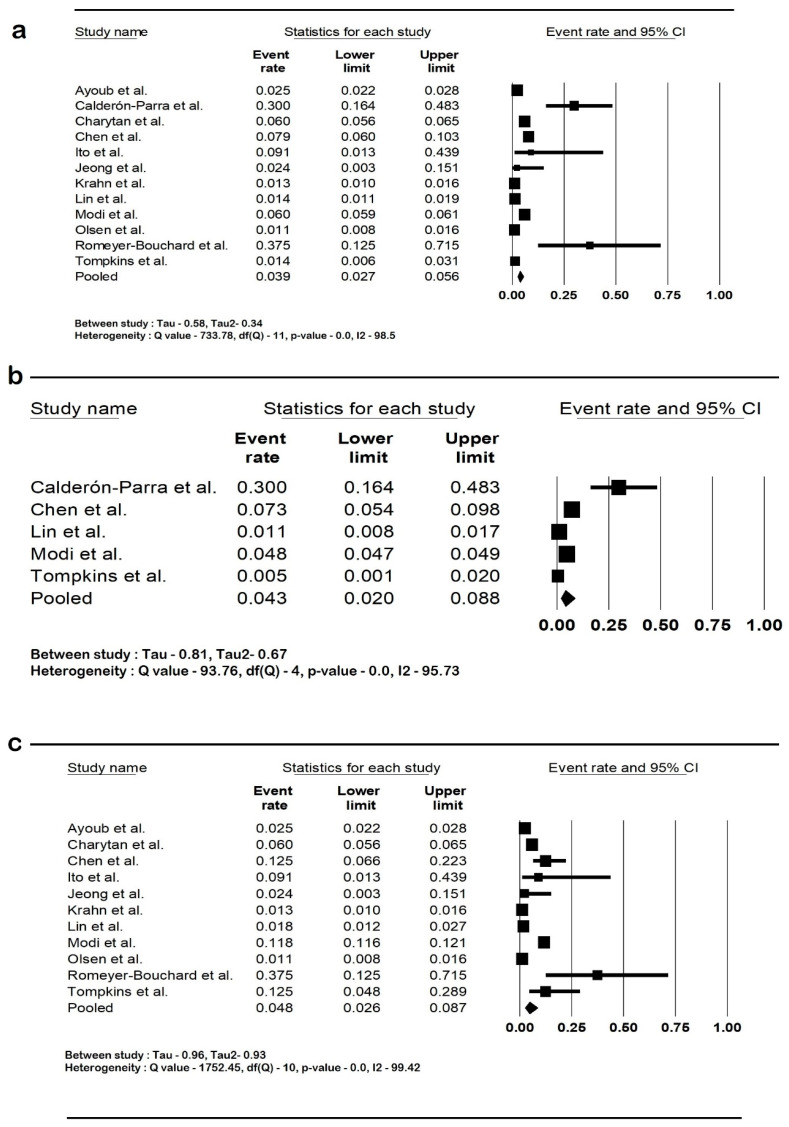
Forest plots of rates of cardiac implantable device infection in (**a**) all renal insufficiency, (**b**) chronic kidney disease, and (**c**) end-stage renal disease. Ayoub et al. [26], Calderón-Parra et al. [28], Charytan et al. [29], Chen et al. [30], Ito et al. [31], Jeong et al. [32], Krahn et al. [40], Lin et al. [33], Modi et al. [1], Olsen et al. [34], Romeyer-Bouchard et al. [36], and Tompkins et al. [37]. Abbreviation: CI, confidence interval.

**Figure 3:**
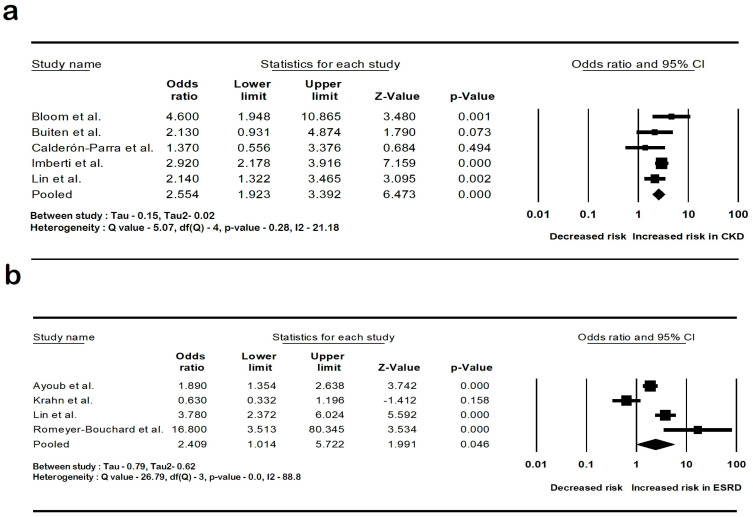
Forest plots of the risk of cardiac implantable device infection in (**a**) chronic kidney disease and (**b**) end-stage renal disease. Ayoub et al. [26], Bloom et al. [8], Buiten et al. [27], Calderón-Parra et al. [28], Imberti et al. [39], Lin et al. [33], and Romeyer-Bouchard et al. [36], Krahn et al. [40]. Abbreviation: CI, confidence interval.

**Figure 4:**
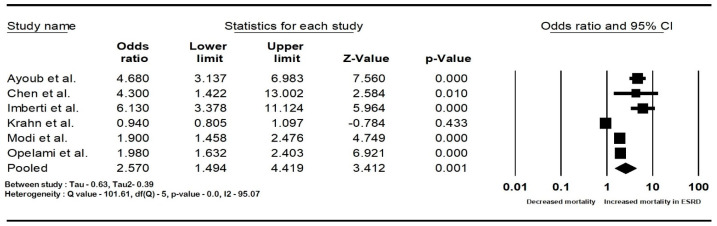
Forest plots of mortality in end-stage renal disease patients with cardiac implantable device infection. Ayoub et al. [26], Chen et al. [30], Imberti et al. [39], Krahn et al. [40], Lin et al. [33], Modi et al. [1], and Opelami et al. [35]. Abbreviation: CI, confidence interval.

The pooled rate of CIED infection in all patients with renal insufficiency with CIED was 3.9% (95% CI: 2.7–5.6; I2: 98.5). The rate of CIED infection among patients with CKD was 4.3% (95% CI: 2–8.8; I2: 95.7), and among patients with ESRD, it was 4.8% (95% CI: 2.6–8.7; I2: 99.4). The forest plots are shown in Figure 2. The pooled risk of CIED infection in the CKD population was OR 2.5 (95% CI: 1.9–3.3; *p* < 0.001; I2: 21.1), and the risk in the ESRD population was OR 2.4 (95% CI: 1.01–5.7; *p* = 0.046; I2: 88.8). The forest plots are shown in Figure 3. ESRD was associated with higher mortality, OR 2.5 (95% CI: 1.4–4.4.8; *p* = 0.001; I2: 95). The pooled mortality results in CKD were not estimated because of there being few studies. The forest plot is shown in Figure 4.

### 3.5. Sensitivity Analysis

Sensitivity analysis was performed by excluding one study at a time to observe if any study had a dominant effect on the effect sizes. There were significant observed changes when the rates of CKD and ESRD were computed. This was due to the large differences in the reported rates of infection. We also repeated all of the analyses, excluding that of Modi et al. [1], from the computations assessing rates of infection, as this cohort had a larger population size compared to others. There was no significant change to the effect sizes while estimating the rates of CIED infections in CKD and ESRD patients when this study was removed. When sensitivity analysis was performed for the pooled odds ratios, a difference was only noted when the risk of ESRD was assessed, and this was likely due to the high odds ratio, 16.8 (95% CI: 3.51–80.2), reported by Romeyer-Bouchard et al. All of the other sensitivity analyses performed did not show any considerable differences. A summary of the sensitivity analysis and forest plots is detailed in the Supplementary Materials, Table S5.

### 3.6. Heterogeneity

Heterogeneity was assessed using the Q statistic and the I2 statistic. When the Q statistic detected heterogeneity, we utilized I2 to quantify the proportion of variability. Most of the analyses had considerable heterogeneity except for the pooled risk of CIED infection in the CKD population, which was low.

### 3.7. Publication Bias

**Figure 5:**
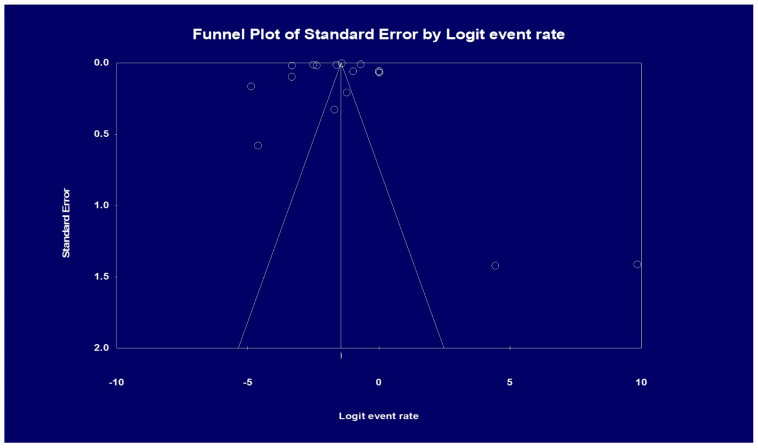
Analysis of publication bias based on funnel plot results. Egger’s test for a regression intercept gave a 1-tailed *p*-value of 0.239, indicating no publication bias. (The intercept (B0) is −8.20, 95% confidence interval (−32.304 and 15.904), with t = 0.725, df = 15).

Visual inspection of the funnel plot showed asymmetry, denoting possible publication bias. Therefore, an Egger’s test was performed, which showed a regression intercept of −8.20 and a 1-tailed test *p*-value of 0.239, indicating that publication bias was absent. The funnel plot with the observed studies is shown in Figure 5.

### 3.8. Quality Assessment

Table 3 shows the quality assessment using the NOS. All of the included studies had a cohort size of >40 patients and presented information about the outcomes of interest. All studies assessed the outcomes and follow-up adequately. All of the studies were scored as high quality.

## 4. Discussion

Our meta-analysis, which included studies from many countries, demonstrates that patients with renal insufficiency are at high risk of developing CIED infections. Patients with ESRD had higher rates of CIED infection and elevated risk of mortality. The pooled infection rates in CKD patients were also higher but less compared to ESRD patients. We also noted that there were considerable differences in the rates of infection reported in different studies.

A previous meta-analysis performed in 2015 by Polyzos et al. involving 26,172 patients showed that ESRD is a risk factor for CIED infections with an OR of 8.87 (95% CI: 3.42–22.3) [41]. Our study showed that the presence of ESRD caused 2.4 times higher odds of infection. This difference could be due to the predominant US population in the previous meta-analysis, whereas our meta-analysis had a more global population. In addition, our meta-analysis also showed that the odds of CIED infection are 2.5 times in CKD, similar to ESRD.

In patients with CKD, their impaired immune system and platelet dysfunction predispose them to developing device infections [42,43]. The risk of bloodstream infections is higher in patients with a central venous catheter than those using an arterio-venous fistula for dialysis [44]. Furthermore, infections can also lead to acute kidney injury that can worsen CKD in patients not on dialysis [45,46]. Other significant risk factors associated with increased infection are prior device infection, corticosteroid use, chronic obstructive pulmonary disease, diabetes mellitus, and oral anticoagulant use. Procedure-related factors such as procedure duration, hematoma, and lead repositioning have also been implicated [14]. Prior CIED infection, chronic skin disease, and steroid use confer the highest risk [45]. Oral anticoagulant use could result in increased post-procedural hematoma formation and increase the risk of infection [14]. Device infections have also been found to be high during the initial months after implantation [33,47]. Also, PPM infections occur at a much lower rate than ICD infections, 1.0 per 1000 device years compared to 8.9 per 1000 device years, respectively [43].

A large study by Modi et al., in their analysis of the NIS from 2011 to 2018, showed that hospital length of stay and total hospital charges increased over time. It was also noted that patients admitted with device infections also experienced more non-infection-related complications, such as pulmonary embolism when compared to admissions for CIED implantation [1]. Calderón-Parra et al. developed a scoring system to risk stratify patients undergoing CIED implantations. The scoring system included variables such as the Charlson index, >2 leads/electrodes, device revisions or replacement, oral anticoagulation, and prior infections. The predictive risk score performed well with an area under the receiver operating characteristic curve of 0.79 (95% CI, 0.71–0.88) in their cohort of 33 patients [28].

Guidelines recommend administering prophylactic antibiotics before the procedure as it is the most effective method for preventing CIED infections. Intravenous cefazolin is administered prior to skin incision, and vancomycin in cases of penicillin allergy [13]. An extra dosage of intraprocedural antibiotics may be warranted if the total duration of antibiotic prophylaxis treatment and procedure time exceed 240 min [48]. Several institutions have “bundled” skin preparation protocols. A bundle component consists of a shower or bath using antibacterial soap [38]. The standard practice involves using 2% chlorhexidine for skin preparation as this is superior to povidone iodine. Evidence of this is derived from other surgical data, but no controlled studies have been performed to assess its efficacy before CIED. During implantation, vigorous irrigation of the pocket is recommended to remove dead tissues [14]. PADIT (Prevention of Arrhythmia Device Infection Trial) compared conventional periprocedural antibiotic and incremental antibiotic use. In the incremental arm, patients received cefazolin and vancomycin together. In addition, either an antibacterial solution was used as a wound pocket wash before closure or oral antibiotics were given post procedure. Although the number of device infections was low, it was not statistically significant. The PADIT study also showed no benefit in local instillation of antibiotics prior to pocket closure [40]. Wu et al. showed that bundled skin antiseptic preparation reduced the incidence of infections from skin flora. This involved using an alcohol solution over the whole chest, povidone-iodine 10 min before the procedure, and then an antiseptic skin preparation [38]. Gram-positive bacteria are the most common causative organism (70–90%), with coagulase-negative staphylococci (CoNS) causing the most infections worldwide [14]. Chen et al. reported that gram-negative bacilli were the most frequent organisms in their cohort [30]. However, despite prophylactic antibiotics, the mortality of patients with CIED infection has increased over the years from 2.9% in 1993 to 4.7% in 2008 [43]. Our study showed that ESRD patients had increased mortality, OR 2.5 (95% CI: 1.49–4.41).

The WRAP-IT study (Worldwide Randomized Antibiotic Envelope Infection Prevention Trial) demonstrated that an antibacterial mesh envelope with minocycline and rifampin reduced the risk of CIED infections in high-risk patients. The rate of infection was lower among patients who received the envelope (0.7% vs. 1.2%) with a hazard ratio of 0.6 (95% CI: 0.3–0.9; *p* = 0.04) [49]. Since patients with CKD and ESRD have an increased risk of infection due to various factors such as immunosuppression and dialysis, the use of antibacterial envelopes could lower the number of infections. If feasible and appropriate, leadless pacemakers are another way to mitigate CIED infections in this high-risk population [14].

Our study has some limitations. First, there may be gaps in information from retrospective studies as they rely on the accuracy of the data that have been collected. Second, retrospective studies may not accurately assess a causal relationship. Third, there was non-uniformity in the method of calculation of GFR among studies evaluating CKD patients. Fourth, we report the rates of CIED infections, which include both pocket and systemic infections, but it is unknown whether the CIED infections are due to one or the other. Fifth, the sensitivity analysis showed that certain studies were outliers as they reported an increased incidence of infection and may have an increased effect on the summary estimate than other studies. Finally, increased heterogeneity was noted in most of our analyses, possibly due to the diverse population and various CKD stages in the studies included.

Despite these limitations, our meta-analysis explores the relationship between renal insufficiency and CIED. To our knowledge, this is the first meta-analysis to report the pooled rates of CIED infections of a large CKD and ESRD population from studies encompassing many countries. Our meta-analysis highlights the importance of risk stratification of CKD and ESRD patients and infection prevention measures to decrease the number of CIED infections.

In the future, more extensive prospective studies are needed to validate these findings and investigate other factors that increase the risk of infection and mortality in patients with renal dysfunction. Additionally, future research is required to determine the rates of pocket and systemic infections in patients with renal insufficiency.

## Figures and Tables

**Table 1:** Summary of the included studies.

Study	Year	Study Years	Study Type	Country	Population Reported	Total Number of Patients with a Cardiac Device, *n*	Total Number of Patients with Renal Insufficiency Included in the Study, *n*(%)
Ayoub et al. [26]	2021	2010–2016	Retrospective, observational, multicenter	USA	ESRD	43,075	14,363(33.3)
Bloom et al. [8]	2006	1999–2004	Retrospective, observational, single center	USA	ESRD and CKD	4856	37(0.7)
Buiten et al. [27]	2014	1996–2012	Prospective, observational, single center	The Netherlands	ESRD and CKD	3147	110(3.4)
Calderón-Parra et al. [28]	2018	2009–2015	Retrospective nested case–control, single center	Spain	CKD	132	33(25)
Charytan et al. [29]	2011	1994–2006	Retrospective observational, multicenter	USA	ESRD	9528	9528(100)
Chen et al. [30]	2020	2012–2017	Retrospective, observational, single center	Taiwan	ESRD and CKD	1237	621(50.2)
Imberti et al. [39]	2023	2016–2020	Prospective, observational, single center	Italy	ESRD and CKD	838	424(50.5)
Ito et al. [31]	2009	1998–2007	Retrospective, observational, single center	Japan	ESRD	71	11(15.4)
Jeong et al. [32]	2018	2001–2017	Retrospective, observational, single center	Korea	ESRD	42	42(100)
Krahn et al. [40]	2018	2012–2015	Cluster randomized cross-over trial, multicenter	Canada and the Netherlands	ESRD and CKD	39,118	6492(16.5)
Lin et al. [33]	2015	1997–2011	Retrospective, observational, population-based cohort study	Taiwan	ESRD and CKD	38,354	3294(8.5)
Modi et al. [1]	2023	2011–2018	Retrospective, observational, population-based cohort study	USA	ESRD and CKD	1,604,173	314,418(19.6)
Olsen et al. [34]	2022	1996–2018	Retrospective, observational, population-based cohort study	Denmark	ESRD	80,430	2829(3.5)
Opelami et al. [35]	2014	2005–2010	Retrospective, observational, population-based cohort study	USA	ESRD	87,798	6665(7.5)
Romeyer-Bouchard et al. [36]	2010	2001–2007	Prospective, observational, single center	France	ESRD	303	3(0.9)
Tompkins et al. [37]	2011	2004–2007	Retrospective, observational, single center	USA	CKD	1440	393(27.2)
Wu et al. [38]	2023	2012–2019	Retrospective, observational, single center	Taiwan	ESRD and CKD	984	492(50)

Abbreviations: CKD—chronic kidney disease; ESRD—end-stage renal disease; USA—United States of America.

**Table 2:** Patient characteristics.

Study	Age, Mean ± SD	Males, *n*(%)	Females, *n*(%)	Total with CIED in ESRD, *n*	Total with CIED in CKD, *n*	Total Number of Patients with Renal Insufficiency and CIED Infection, *n*(%)
Ayoub et al. [26]	64.57 ± 0.23	9999(69.7)	4364(30.3)	14363	NR	357(2.4)
Bloom et al. [8]	NR	NR	NR	5	32	37(100)
Buiten et al. [27]	71 ± 10	75(68.1)	35(31.9)	17	93	3(2.7)
Calderón-Parra et al. [28]	65.5 ± 16.7	NR	NR	NR	33	9(27.2)
Charytan et al. [29]	64.5 ± 12	6652(69.8)	2876(30.2)	9528	NR	574(6)
Chen et al. [30]	72.5 ± 12	NR	NR	72	549	49(7.8)
Imberti et al. [39]	76 ± 10.5	NR	NR	23	401	NR
Ito et al. [31]	59 ± 3	9(82)	2(18)	11	NR	1(9)
Jeong et al. [32]	62.3 ± 34.8	23(55)	19(45)	42	NR	1(2.3)
Krahn et al. [40]	72 ± 13	2345(36.1)	4174(64.2)	6492	NR	82(1.2)
Lin et al. [33]	70 ± 9.9	1627(49.3)	1667(50.7)	1367	1927	47(1.4)
Modi et al. [1]	67	NR	NR	54,542	259,876	18,888(6)
Olsen et al. [34]	NR	NR	NR	2829	NR	32(1.1)
Opelami et al. [35]	NR	4262(63.9)	2403(36.1)	6665	NR	6665(100)
Romeyer-Bouchard et al. [36]	66 ± 11	NR	NR	8	NR	3(37.5)
Tompkins et al. [37]	66.4 ± 11.3	222(56.4)	171(43.6)	32	361	6(1.5)
Wu et al. [38]	75 ± 10	247(50.2)	245(49.8)	46	446	5(1.01)

Abbreviations: CKD—chronic kidney disease; CIED—cardiac implantable electronic device; ESRD—end-stage renal disease; NR—not reported; SD—standard deviation.

**Table 3:** Quality assessment of studies based on the Newcastle—Ottawa quality assessment form.

Study	Selection				Comparability	Outcome			Score	Quality
	Representativeness of the Average Adult in the Community	Cohort Size	Information on Outcomes	Outcome not Present at Start	Additional Intervention	Adequate Assessment	Follow up Time	Adequacy of Follow-Up	Max = 7; High > 5; Medium 3–5; Low < 3	
	Population Based: 1; Multi Center: 0.5; Single Center: 0	>40 Patients: 1; 39 to 20: 0.5; <20: 0	Information with Clarity: 1; Information Derived: 0.5	Not Present: 1; Present: 0	Yes: 1; No: 0	Yes: 1; No: 0	Yes: 1; Not Mentioned: 0	All Patients Followed up: 1; >50% Followed up: 0.5; <50% Followed up OR Not Mentioned: 0		
Ayoub et al. [26]	★	★	★	●	★	★	●	★	6	High
Bloom et al. [8]	●	★	★	●	★	★	●	★	5	High
Buiten et al. [27]	●	★	★	●	●	★	★	★	5	High
Calderón-Parra et al. [28]	●	★	★	★	★	★	★	★	7	High
Charytan et al. [29]	★	★	★	●	●	★	★	★	6	High
Chen et al. [30]	●	★	★	●	★	★	★	★	6	High
Imberti et al. [39]	●	★	★	●	●	★	★	★	5	High
Ito et al. [31]	●	★	★	●	★	★	★	★	6	High
Jeong et al. [32]	●	★	★	●	●	★	★	★	5	High
Krahn et al. [40]	☆	★	★	★	★	★	★	★	7.5	High
Lin et al. [33]	★	★	★	●	●	★	●	★	5	High
Modi et al. [1]	★	★	★	●	●	★	●	★	5	High
Olsen et al. [34]	★	★	★	●	●	★	●	★	5	High
Opelami et al. [35]	★	★	★	●	●	★	●	★	5	High
Romeyer-Bouchard et al. [36]	●	★	★	●	●	★	★	★	5	High
Tompkins et al. [37]	●	★	★	●	★	★	●	★	5	High
Wu et al. [38]	●	★	★	●	★	★	★	★	6	High

★ 1 point; ☆ 0.5 point; ● 0 point.

## Supplementary Materials

The following supporting information can be downloaded at: https://www.mdpi.com/article/10.3390/diseases12100247/s1. Table S1: Search strategy; Table S2: Studies with overlapping cohorts; Table S3: MOOSE Checklist for Meta-analyses of Observational Studies; Table S4: Preferred Reporting Items for Systematic Reviews and Meta-Analyses: The PRISMA Statement; Table S5: Summary of Sensitivity Analysis; Figure S1: Forest plot of sensitivity analysis of rates of cardiac implantable device infection in renal insufficiency; Figure S2: Forest plot of sensitivity analysis of rates of cardiac implantable device infection in renal insufficiency excluding Modi et al.; Figure S3: Forest plot of sensitivity analysis of rates of cardiac implantable device infection in CKD; Figure S4: Forest plot of sensitivity analysis of rates of cardiac implantable device infection in CKD without Modi et al.; Figure S5: Forest plot of sensitivity analysis of rates of cardiac implantable device infection in ESRD; Figure S6: Forest plot of sensitivity analysis of rates of cardiac implantable device infection in ESRD without Modi et al.; Figure S7: Forest plot of sensitivity analysis of the risk of cardiac implantable device infection in CKD; Figure S8: Forest plot of sensitivity analysis of the risk of cardiac implantable device infection in ESRD; Figure S9: Forest plot of sensitivity analysis of risk of mortality in ESRD due to cardiac implantable device infection.
