# Supplementary material for: Cardiac Implantable Electronic Device Infections in Patients with Renal Insufficiency: A Systematic Review and Meta-Analysis
Source: Diseases. 2024 Oct 10;12(10):247. doi: 10.3390/diseases12100247 (PMC11507648; doi:10.3390/diseases12100247)

Supplementary materials: Cardiac Implantable Electronic Device Infections in Patients with Renal Insufficiency: A Systematic Review and Meta-Analysis

Deepak Chandramohan [1,*], Prabhat Singh [2], Hari Naga Garapati [3], Raghunandan Konda [1], Divya Chandramohan [4], Nihar Jena [5], Atul Bali [6] and Prathap Kumar Simhadri [7]

Table S1. Search strategy.

Digital database	Search strategy	Search results
PubMed	(("Cardiac Implantable Electronic Devices" OR "CIED" OR "pacemakers" OR "implantable cardioverter-defibrillators" OR "ICDs" OR "ICD" OR "pacemaker" or "cardiac implantable electronic device") AND ("infections" OR "infection" OR "complications" OR "complication") AND ("incidence" OR "prevalence" OR "clinical outcomes" OR "clinical outcome" OR "morbidity" OR "mortality" OR "risk factors" OR "risk factor" OR "management" AND ("End-Stage Renal Disease" OR "ESRD" OR "end stage renal disease" OR "Chronic kidney disease" OR CKD)).	436
EMBASE	('cardiac implantable electronic devices'/exp OR 'CIED' OR 'pacemaker'/exp OR 'implantable cardioverter defibrillator'/exp OR 'ICD' OR 'pacemakers' OR 'implantable cardioverter-defibrillators' OR 'cardiac implantable electronic device') AND ('infection'/exp OR 'infections' OR 'complication'/exp OR 'complications') AND ('incidence'/exp OR 'prevalence'/exp OR 'clinical outcome'/exp OR 'clinical outcomes' OR 'morbidity'/exp OR 'mortality'/exp OR 'risk factor'/exp OR 'risk factors' OR 'management'/exp) AND ('end stage renal disease'/exp OR 'ESRD' OR 'chronic kidney disease'/exp OR 'CKD')	2866
Cochrane Library	("Cardiac Implantable Electronic Devices" OR "CIED" OR "pacemakers" OR "implantable cardioverter-defibrillators" OR "ICDs" OR "ICD" OR "pacemaker" OR "cardiac implantable electronic device") AND ("infections" OR "infection" OR "complications" OR "complication") AND ("incidence" OR "prevalence" OR "clinical outcomes" OR "clinical outcome" OR "morbidity" OR "mortality" OR "risk factors" OR "risk factor" OR "management") AND ("End-Stage Renal Disease" OR "ESRD" OR "end stage renal disease" OR "Chronic kidney disease" OR "CKD")	40
Google Scholar	"Cardiac device infections" "Chronic kidney disease" OR "End stage renal disease"	296

Table S2. Studies with overlapping cohorts.

Year published	Study years	Study	Overlap with
2007	1998-2005	Dasgupta, A., Montalvo, J., Medendorp, S., Lloyd-Jones, D. M., Ghossein, C., Goldberger, J. J., & Passman, R. (2007). Increased complication rates of cardiac rhythm management devices in ESRD patients. *American Journal of Kidney Diseases*, *49*(5), 656–663. https://doi.org/10.1053/j.ajkd.2007.02.272	Bloom et al.
2009	2000-2007	Lekkerkerker, J. C., Van Nieuwkoop, C., Trines, S. A., Van Der Bom, J. G., Bernards, A. T., Van De Velde, E. T., Bootsma, M., Zeppenfeld, K., Jukema, J. W., Borleffs, J. W., Schalij, M. J., & Van Erven, L. (2009). Risk factors and time delay associated with cardiac device infections: Leiden device registry. *Heart*, *95*(9), 715–720. https://doi.org/10.1136/hrt.2008.151985	Buiten et al.
2010	1991-2008	Le, K. Y., Sohail, M. R., Friedman, P. A., Uslan, D. Z., Stephen, S., Hayes, D. L., Wilson, W. R., Steckelberg, J. M., & Baddour, L. M. (2011). Clinical Predictors of Cardiovascular Implantable Electronic Device-Related Infective Endocarditis. *Pacing and Clinical Electrophysiology*, *34*(4), 450–459. https://doi.org/10.1111/j.1540-8159.2010.02991.x	Tompkins et al.
2011	1991-2008	Sohail, M. R., Hussain, S., Le, K. Y., Dib, C., Lohse, C. M., Friedman, P. A., Hayes, D. L., Uslan, D. Z., Wilson, W. R., Steckelberg, J. M., & Baddour, L. M. (2011). Risk factors associated with early- versus late-onset implantable cardioverter-defibrillator infections. *Journal of Interventional Cardiac Electrophysiology*, *31*(2), 171–183. https://doi.org/10.1007/s10840-010-9537-x	Le et al and Tompkins et al.
2011	1993-2008	Greenspon, A. J., Patel, J., Lau, E., Ochoa, J., Frisch, D. R., Ho, R. T., Pavri, B. B., & Kurtz, S. M. (2011). 16-Year trends in the infection burden for pacemakers and implantable Cardioverter-Defibrillators in the United States. *Journal of the American College of Cardiology*, *58*(10), 1001–1006. https://doi.org/10.1016/j.jacc.2011.04.033	Opelami et al.
2014	1991-2008	Hickson, L. T. J., Gooden, J. Y., Le, K. Y., Baddour, L. M., Friedman, P. A., Hayes, D. L., Wilson, W. R., Steckelberg, J. M., & Sohail, M. R. (2014). Clinical presentation and outcomes of cardiovascular implantable electronic device infections in hemodialysis patients. *American Journal of Kidney Diseases*, *64*(1), 104–110. https://doi.org/10.1053/j.ajkd.2013.11.018	Le et al and Tompkins et al.
2015	2005-2009	Guha, A., Maddox, W. R., Colombo, R. E., Nahman, N. S., Kintziger, K. W., Waller, J. L., Diamond, M., Murphy, M., Kheda, M., Litwin, S. E., & Sorrentino, R. (2015). Cardiac implantable electronic device infection in patients with end-stage renal disease. *Heart Rhythm*, *12*(12), 2395–2401. https://doi.org/10.1016/j.hrthm.2015.08.003	Charytan et al.
2015	2007	Sohail, M. R., Henrikson, C. A., Braid-Forbes, M. J., Forbes, K. F., & Lerner, D. J. (2014). Increased Long-Term Mortality in Patients with Cardiovascular Implantable Electronic Device Infections. *Pacing and Clinical Electrophysiology*, *38*(2), 231–239. https://doi.org/10.1111/pace.12518	Ayoub et al.
2020	2012-2018	Pun, P. H., Parzynski, C. S., Friedman, D. J., Sanders, G. D., Curtis, J. P., & Al-Khatib, S. M. (2020). Trends in Use and In-Hospital Outcomes of subcutaneous Implantable cardioverter defibrillators in patients undergoing Long-Term Dialysis. *Clinical Journal of the American Society of Nephrology*, *15*(11), 1622–1630. https://doi.org/10.2215/cjn.07920520	Modi et al.
2020	2016	Rennert-May, E., Chew, D. S., Lu, S., Chú, Á., Kuriachan, V., & Somayaji, R. (2020). Epidemiology of cardiac implantable electronic device infections in the United States: A population-based cohort study. *Heart Rhythm*,	Ayoub et al.

		17(7), 1125–1131. https://doi.org/10.1016/j.hrthm.2020.02.012	
2022	2016-2018	El-Chami, M. F., Knight, B. P., Liu, Y., Brisben, A. J., Sohail, M. R., & Griffiths, R. I. (2022). Temporal trends of device-related infection in de novo transvenous implantable cardioverter-defibrillator Medicare patients with underlying kidney disease. *Heart Rhythm*, *19*(10), 1689–1695. https://doi.org/10.1016/j.hrthm.2022.05.022	Modi et al.
2022	2013-2016	Gold, M. R., Aasbo, J. D., Weiss, R., Burke, M. C., Gleva, M. J., Knight, B. P., Miller, M. A., Schuger, C., Carter, N., Leigh, J., Brisben, A. J., & El-Chami, M. F. (2022). Infection in patients with subcutaneous implantable cardioverter-defibrillator: Results of the S-ICD Post Approval Study. *Heart Rhythm*, *19*(12), 1993–2001. https://doi.org/10.1016/j.hrthm.2022.07.031	Modi et al.
2023	2012-2020	Kalot, M., Bahuva, R., Pandey, R., Farooq, W., Mir, A., Khan, A., Kerling, D., Aftab, H., Kovacs, A., Gupta, S., Smith, M., Tian, L., Amuthan, R., & Sharma, U. C. (2023). Risk factors associated with higher mortality in patients with cardiac implantable electronic device infection. *Journal of Cardiovascular Electrophysiology*, *34*(3), 738–747. https://doi.org/10.1111/jce.15817	Ayoub et al.

Table S3. MOOSE Checklist for Meta-analyses of Observational Studies.

Item No	Recommendation	Reported on Page No
	Reporting of background should include	
1	Problem definition	2,4
2	Hypothesis statement	-
3	Description of study outcome(s)	6
4	Type of exposure or intervention used	6
5	Type of study designs used	6,7
6	Study population	4,6
	Reporting of search strategy should include	
7	Qualifications of searchers (eg, librarians and investigators)	5, Title page
8	Search strategy, including time period included in the synthesis and key words	5, Table 1
9	Effort to include all available studies, including contact with authors	5,6
10	Databases and registries searched	5, Supplementary data
11	Search software used, name and version, including special features used (eg, explosion)	5
12	Use of hand searching (eg, reference lists of obtained articles)	5
13	List of citations located and those excluded, including justification	8, Figure 1, Supplementary data
14	Method of addressing articles published in languages other than English	6
15	Method of handling abstracts and unpublished studies	6
16	Description of any contact with authors	-
	Reporting of methods should include	
17	Description of relevance or appropriateness of studies assembled for assessing the hypothesis to be tested	5,6
18	Rationale for the selection and coding of data (eg, sound clinical principles or convenience)	6
19	Documentation of how data were classified and coded (eg, multiple raters, blinding and inter-rater reliability)	5,6
20	Assessment of confounding (eg, comparability of cases and controls in studies where appropriate)	7
21	Assessment of study quality, including blinding of quality assessors, stratification or regression	7

	on possible predictors of study results	
22	Assessment of heterogeneity	6
23	Description of statistical methods (eg, complete description of fixed or random effects models, justification of whether the chosen models account for predictors of study results, dose-response models, or cumulative meta-analysis) in sufficient detail to be replicated	6,7
24	Provision of appropriate tables and graphics	Figures 2-5, Tables 1-3
	Reporting of results should include	
25	Graphic summarizing individual study estimates and overall estimate	8-11, Figure 2-5
26	Table giving descriptive information for each study included	Table 1,2
27	Results of sensitivity testing (eg, subgroup analysis)	10, Supplementary data
28	Indication of statistical uncertainty of findings	13,14
	Reporting of discussion should include	
29	Quantitative assessment of bias (eg, publication bias)	12, Fig 2
30	Justification for exclusion (eg, exclusion of non-English language citations)	6
31	Assessment of quality of included studies	11, Figure 5
	Reporting of conclusions should include	
32	Consideration of alternative explanations for observed results	13,14
33	Generalization of the conclusions (ie, appropriate for the data presented and within the domain of the literature review)	13,14
34	Guidelines for future research	14
35	Disclosure of funding source	15

From: Stroup DF, Berlin JA, Morton SC, et al, for the Meta-analysis Of Observational Studies in Epidemiology (MOOSE) Group. Meta-analysis of Observational Studies in Epidemiology. A Proposal for Reporting. *JAMA*. 2000;283(15):2008-2012. doi: 10.1001/jama.283.15.2008.

Table S4. Preferred Reporting Items for Systematic Reviews and Meta-Analyses: The PRISMA Statement.

Section/topic	#	Checklist item	Reported on page #
		**TITLE**	
Title	1	Identify the report as a systematic review, meta-analysis, or both.	1
		**ABSTRACT**	
Structured summary	2	Provide a structured summary including, as applicable: background; objectives; data sources; study eligibility criteria, participants, and interventions; study appraisal and synthesis methods; results; limitations; conclusions and implications of key findings; systematic review registration number.	2
		**INTRODUCTION**	
Rationale	3	Describe the rationale for the review in the context of what is already known.	4,5
Objectives	4	Provide an explicit statement of questions being addressed with reference to participants, interventions, comparisons, outcomes, and study design (PICOS).	4,5
		**METHODS**	
Protocol and registration	5	Indicate if a review protocol exists, if and where it can be accessed (e.g., Web address), and, if available, provide registration information including registration number.	6
Eligibility criteria	6	Specify study characteristics (e.g., PICOS, length of follow-up) and report characteristics (e.g., years considered, language, publication status) used as criteria for eligibility, giving rationale.	5,6
Information sources	7	Describe all information sources (e.g., databases with dates of coverage, contact with study authors to identify additional studies) in the search and date last searched.	5, Supplementary information
Search	8	Present full electronic search strategy for at least one database, including any limits used, such that it could be repeated.	5, Figure 1, Supplementary information 40
Study selection	9	State the process for selecting studies (i.e., screening, eligibility, included in	5,6

		systematic review, and, if applicable, included in the meta-analysis).	
Data collection process	10	Describe method of data extraction from reports (e.g., piloted forms, independently, in duplicate) and any processes for obtaining and confirming data from investigators.	6
Data items	11	List and define all variables for which data were sought (e.g., PICOS, funding sources) and any assumptions and simplifications made.	6
Risk of bias in individual studies	12	Describe methods used for assessing risk of bias of individual studies (including specification of whether this was done at the study or outcome level), and how this information is to be used in any data synthesis.	7,8
Summary measures	13	State the principal summary measures (e.g., risk ratio, difference in means).	6,7
Synthesis of results	14	Describe the methods of handling data and combining results of studies, if done, including measures of consistency (e.g., I^2) for each meta-analysis.	7
Risk of bias across studies	15	Specify any assessment of risk of bias that may affect the cumulative evidence (e.g., publication bias, selective reporting within studies).	7,8
Additional analyses	16	Describe methods of additional analyses (e.g., sensitivity or subgroup analyses, meta-regression), if done, indicating which were pre-specified.	10
**RESULTS**			
Study selection	17	Give numbers of studies screened, assessed for eligibility, and included in the review, with reasons for exclusions at each stage, ideally with a flow diagram.	8, Figure 1
Study characteristics	18	For each study, present characteristics for which data were extracted (e.g., study size, PICOS, follow-up period) and provide the citations.	Table 1
Risk of bias within studies	19	Present data on risk of bias of each study and, if available, any outcome level assessment (see item 12).	11, Table 3
Results of individual studies	20	For all outcomes considered (benefits or harms), present, for each study: (a) simple summary data for each intervention group (b) effect estimates and confidence intervals, ideally with a forest plot.	Table 2
Synthesis of results	21	Present the main results of the review. If meta-analyses are done, include for each, confidence intervals and measures of consistency	9,10, Figure 2-4
Risk of bias across studies	22	Present results of any assessment of risk of bias across studies (see Item 15).	11, Figure 5
Additional analysis	23	Give results of additional analyses, if done (e.g., sensitivity or subgroup analyses, meta-regression [see Item 16]).	10, Supplementary information
**DISCUSSION**			
Summary of evidence	24	Summarize the main findings including the strength of evidence for each main outcome; consider their relevance to key groups (e.g., healthcare providers, users, and policy makers).	11, 12
Limitations	25	Discuss limitations at study and outcome level (e.g., risk of bias), and at review-level (e.g., incomplete retrieval of identified research, reporting bias).	13, 14
Conclusions	26	Provide a general interpretation of the results in the context of other evidence, and implications for future research.	14
**FUNDING**			
Funding	27	Describe sources of funding for the systematic review and other support (e.g., supply of data); role of funders for the systematic review.	15

From: Moher D, Liberati A, Tetzlaff J, Altman DG, The PRISMA Group (2009). Preferred Reporting Items for Systematic Reviews and Meta-Analyses: The PRISMA Statement. PLoS Med 6(7): e1000097. doi:10.1371/journal.pmed1000097.

For more information, visit: **www.prisma-statement.org**.

Table S5. Summary of Sensitivity Analysis.

Summary effect size	Sensitivity analysis result
Rates of CIED infection in renal insufficiency	Significant
Rates of CIED infection in CKD	Significant
Rates of CIED infection in ESRD	Significant
Rates of CIED infection in renal insufficiency after removing Modi et al.	Not significant
Rates of CIED infection in CKD after removing Modi et al.	Not significant
Rates of CIED infection in ESRD after removing Modi et al.	Not significant
Risk of CIED infection in CKD	Not significant
Risk of CIED infection in ESRD	Significant
Mortality in ESRD due to CIED infection	Not significant

The forest plots for the sensitivity analysis are shown below in supplementary figures 1-8.

Figure S1. - Forest plot of sensitivity analysis of rates of cardiac implantable device infection in renal insufficiency. The sensitivity analysis was significant. The changes to the individual studies are shown next to the studies.

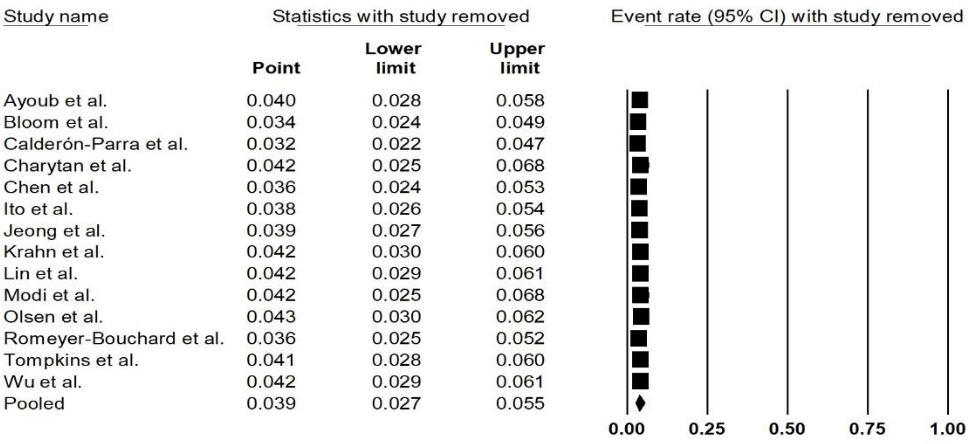

Figure S2. Forest plot of sensitivity analysis of rates of cardiac implantable device infection in renal insufficiency excluding Modi et al. The point estimate did not significantly change. The changes to the individual studies are shown next to the studies.

Study name	Statistics for each study		
	**Event rate**	**Lower limit**	**Upper limit**
Ayoub et al.	0.025	0.022	0.028
Calderón-Parra et al.	0.300	0.164	0.483
Charytan et al.	0.060	0.056	0.065
Chen et al.	0.079	0.060	0.103
Ito et al.	0.091	0.013	0.439
Jeong et al.	0.024	0.003	0.151
Krahn et al.	0.013	0.010	0.016
Lin et al.	0.014	0.011	0.019
Olsen et al.	0.011	0.008	0.016
Romeyer-Bouchard et al.	0.375	0.125	0.715
Tompkins et al.	0.014	0.006	0.031
Pooled	0.039	0.023	0.065

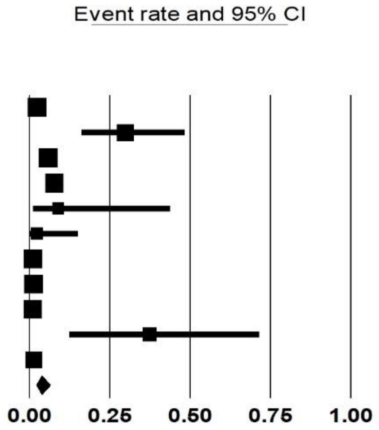

Figure S3. Forest plot of sensitivity analysis of rates of cardiac implantable device infection in CKD. The sensitivity analysis was significant. The changes to the individual studies are shown next to the studies.

Study name	Statistics with study removed		
	**Point**	**Lower limit**	**Upper limit**
Calderón-Parra et al.	0.030	0.015	0.058
Chen et al.	0.041	0.014	0.116
Lin et al.	0.066	0.034	0.123
Modi et al.	0.047	0.012	0.168
Tompkins et al.	0.058	0.027	0.121
Pooled	0.046	0.023	0.091

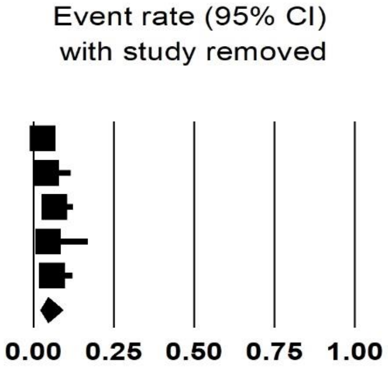

Figure S4 - Forest plot of sensitivity analysis of rates of cardiac implantable device infection in CKD without Modi et al. The changes to the individual studies are shown next to the studies. There were no significant changes to the point estimate.

Study name	Statistics for each study		
	Event rate	Lower limit	Upper limit
Calderón-Parra et al.	0.300	0.164	0.483
Chen et al.	0.073	0.054	0.098
Lin et al.	0.011	0.008	0.017
Tompkins et al.	0.005	0.001	0.020
Pooled	0.038	0.008	0.157

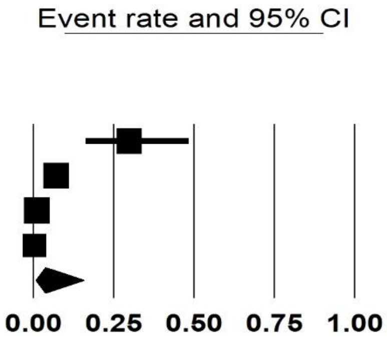

Figure S5. Forest plot of sensitivity analysis of rates of cardiac implantable device infection in ESRD. The sensitivity analysis was significant. The changes to the individual studies are shown next to the studies.

Study name	Statistics with study removed		
	Point	Lower limit	Upper limit
Ayoub et al.	0.051	0.029	0.090
Charytan et al.	0.049	0.021	0.109
Chen et al.	0.043	0.023	0.081
Ito et al.	0.047	0.025	0.085
Jeong et al.	0.050	0.027	0.092
Krahn et al.	0.056	0.031	0.099
Lin et al.	0.054	0.029	0.099
Modi et al.	0.041	0.024	0.069
Olsen et al.	0.057	0.031	0.103
Romeyer-Bouchard et al.	0.040	0.022	0.075
Tompkins et al.	0.044	0.023	0.082
Pooled	0.048	0.026	0.087

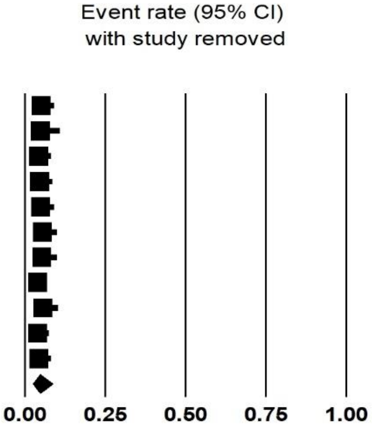

Figure S6. - Forest plot of sensitivity analysis of rates of cardiac implantable device infection in ESRD without Modi et al. There were no significant changes to the point estimate. The changes to the individual studies are shown next to the studies.

Study name	Statistics for each study		
	Event rate	Lower limit	Upper limit
Ayoub et al.	0.025	0.022	0.028
Buiten et al.	0.972	0.678	0.998
Charytan et al.	0.060	0.056	0.065
Chen et al.	0.125	0.066	0.223
Ito et al.	0.091	0.013	0.439
Jeong et al.	0.024	0.003	0.151
Krahn et al.	0.013	0.010	0.016
Lin et al.	0.018	0.012	0.027
Olsen et al.	0.011	0.008	0.016
Romeyer-Bouchard et al.	0.375	0.125	0.715
Tompkins et al.	0.125	0.048	0.289
Pooled	0.050	0.029	0.084

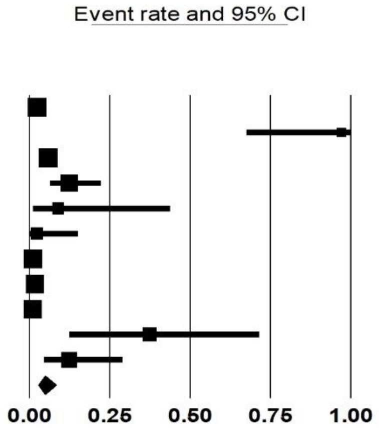

Figure S7: Forest plot of sensitivity analysis of the risk of cardiac implantable device infection in CKD. There were no significant changes to the point estimate. The changes to the individual studies are shown next to the studies.

Study name	Statistics with study removed				
	Point	Lower limit	Upper limit	Z-Value	p-Value
Bloom et al.	2.465	1.902	3.196	6.815	0.000
Buiten et al.	2.581	1.823	3.654	5.345	0.000
Calderón-Parra et al.	2.741	2.176	3.454	8.556	0.000
Imberti et al.	2.286	1.513	3.454	3.925	0.000
Lin et al.	2.669	1.831	3.890	5.109	0.000
Pooled	2.554	1.923	3.392	6.473	0.000

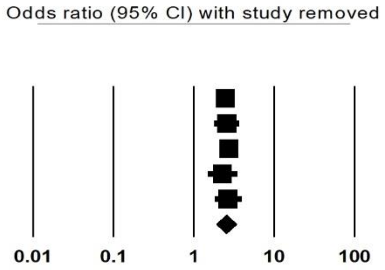

Figure S8. - Forest plot of sensitivity analysis of the risk of cardiac implantable device infection in ESRD. The sensitivity analysis was significant. The changes to the individual studies are shown next to the studies.

Study name	Statistics with study removed				
	Point	Lower limit	Upper limit	Z-Value	p-Value
Ayoub et al.	3.004	0.631	14.311	1.381	0.167
Krahn et al.	3.611	1.612	8.090	3.119	0.002
Lin et al.	2.139	0.647	7.071	1.246	0.213
Romeyer-Bouchard et al.	1.701	0.730	3.961	1.231	0.218
Pooled	2.409	1.014	5.722	1.991	0.046

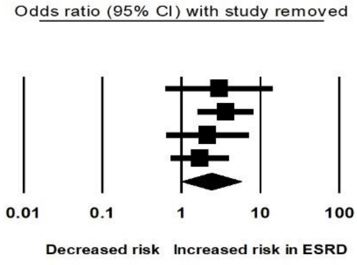

Figure S9 - Forest plot of sensitivity analysis of risk of mortality in ESRD due to cardiac implantable device infection. There were no significant changes to the point estimate. The changes to the individual studies are shown next to the studies.

Study name	Statistics with study removed				
	Point	Lower limit	Upper limit	Z-Value	p-Value
Ayoub et al.	2.225	1.302	3.803	2.924	0.003
Chen et al.	2.415	1.364	4.279	3.023	0.003
Imberti et al.	2.168	1.256	3.743	2.779	0.005
Krahn et al.	3.144	2.009	4.918	5.015	0.000
Modi et al.	2.796	1.411	5.540	2.946	0.003
Opelami et al.	2.799	1.323	5.923	2.691	0.007
Pooled	2.570	1.494	4.419	3.412	0.001

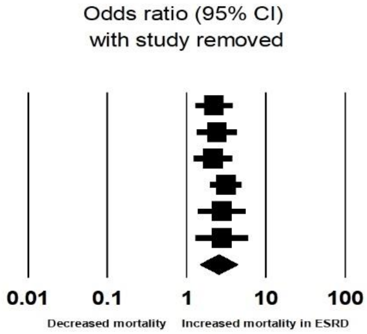

Supplement: Supplementary file 1 [file diseases-12-00247-s001.zip › diseases-3163464-supplementary.pdf]
